# An Investigation into the Etiological Agents of Swine Dysentery in Australian Pig Herds

**DOI:** 10.1371/journal.pone.0167424

**Published:** 2016-12-01

**Authors:** Tom La, Nyree D. Phillips, David J. Hampson

**Affiliations:** School of Veterinary and Life Sciences, Murdoch University, Murdoch, Western Australia, Australia; Animal and Plant Health Agency, UNITED KINGDOM

## Abstract

Swine dysentery (SD) is a mucohemorrhagic colitis, classically seen in grower/finisher pigs and caused by infection with the anaerobic intestinal spirochete *Brachyspira hyodysenteriae*. More recently, however, the newly described species *Brachyspira hampsonii* and *Brachyspira suanatina* have been identified as causing SD in North America and/or Europe. Furthermore, there have been occasions where strains of *B*. *hyodysenteriae* have been recovered from healthy pigs, including in multiplier herds with high health status. This study investigated whether cases of SD in Australia may be caused by the newly described species; how isolates of *B*. *hyodysenteriae* recovered from healthy herds compared to isolates from herds with disease; and how contemporary isolates compare to those recovered in previous decades, including in their plasmid gene content and antimicrobial resistance profiles. In total 1103 fecal and colon samples from pigs in 97 Australian herds were collected and tested. Of the agents of SD only *B*. *hyodysenteriae* was found, being present in 34 (35.1%) of the herds, including in 14 of 24 (58%) herds that had been considered to be free of SD. Multilocus sequence typing applied to 96 isolates from 30 herds and to 53 Australian isolates dating from the 1980s through the early 2000s showed that they were diverse, distinct from those reported in other countries, and that the 2014/16 isolates generally were different from those from earlier decades. These findings provided evidence for ongoing evolution of *B*. *hyodysenteriae* strains in Australia. In seven of the 20 herds where multiple isolates were available, two to four different sequence types (STs) were identified. Isolates with the same STs also were found in some herds with epidemiological links. Analysis of a block of six plasmid virulence-associated genes showed a lack of consistency between their presence or absence and their origin from herds currently with or without disease; however, significantly fewer isolates from the 2000s and from 2014/16 had this block of genes compared to isolates from the 1980s and 1990s. It is speculated that loss of these genes may have been responsible for the occurrence of milder disease occurring in recent years. In addition, fewer isolates from 2014/16 were susceptible to the antimicrobials lincomycin, and to a lesser extent tiamulin, than those from earlier Australian studies. Four distinct multi-drug resistant strains were identified in five herds, posing a threat to disease control.

## Introduction

Swine dysentery (SD) typically manifests as a severe mucohemorrhagic colitis arising from infection of the caecum and colon of grower-finisher pigs with the anaerobic intestinal spirochete *Brachyspira hyodysenteriae* [1]. SD can severely depress feed conversion efficiency, may require considerable antimicrobial use for its control, and represents an animal welfare issue. Strains of *B*. *hyodysenteriae* that are resistant to antimicrobial agents increasing have been recorded, particularly in Europe. This classical form of SD re-emerged as a problem in North America towards the end of the first decade of the 21st century, after being largely absent for 10–15 years, whilst the disease has remained endemic in most other pig producing regions of the world.

Until recently *B*. *hyodysenteriae* was believed to be the only causative agent of SD; however, since around 2007 outbreaks of bloody diarrhea indistinguishable from SD have been documented in Canada and the USA in herds where *B*. *hyodysenteriae* could not be identified. Investigation of these cases led to the recognition of a novel, strongly beta-hemolytic *Brachyspira* species that recently has been named *Brachyspira hampsonii* [2,3]. Experimental inoculations of pigs have established the pathogenic potential of this new species [4–6]. Concurrently, cases of SD caused by *B*. *hampsonii* were recorded in pigs in Europe, and strains of the species were isolated from migratory water birds in Canada and in Spain [7–10]. A distinct strongly beta-hemolytic species named *Brachyspira suanatina* that causes an SD-like disease in pigs also has been identified in feral water birds and pigs in Scandinavia [11,12]. In the same time frame interest has increased in strains of *B*. *hyodysenteriae* that may have reduced capacity to colonize and cause disease, since strains of this sort can complicate the diagnosis and control of SD [13,14]. In Australia, *B*. *hyodysenteriae* has been isolated from apparently healthy herds that gave what were initially thought to be false positive results in a serological ELISA [15].

The re-emergence of *Brachyspira* spp., including antimicrobial resistant strains of *B*. *hyodysenteriae* and novel species like *B*. *hampsonii* as pathogens, has re-ignited significant concerns for pork-producers worldwide. The aims of this project were to determine whether novel pathogenic *Brachyspira* species were present but unrecognized in Australian pig herds; to investigate the distribution, diversity, antimicrobial susceptibility and virulence-associated plasmid gene content of contemporary isolates of *B*. *hyodysenteriae* from Australian herds, including those with clinical SD, no disease or with uncertain health status; and to compare these attributes with those for Australian isolates recovered in earlier decades.

## Materials and Methods

### Permissions

This study was conducted with the approval of the Murdoch University Animal Ethics Committee under permit number R2292.

### Sample collection

During the period June 2014 to June 2016 a total of 1103 diagnostic samples, comprising 606 faecal and 497 colon samples that had been collected from grower/finisher pigs in 97 pig herds in Australia were submitted for testing by consulting pig veterinarians and through veterinary diagnostic laboratories. Twenty-four of these herds were considered by their veterinarians to be free of SD, and for this study had additional samples taken at the abattoir during routine screening, including samples of the colonic mucosa and faeces from pigs that did not show clinical signs. The other samples were from pigs in 25 herds where SD was believed likely to be present based on past history or the clinical signs in the pigs sampled, and from 48 herds where the health status was unknown, but where diarrhea and/or mild colitis had been identified and/or the veterinarian wanted to exclude the possibility of SD. The 97 herds were located in five Australian States: Victoria (n = 45), South Australia (n = 21), Western Australia (n = 13), Queensland (n = 11), and New South Wales (n = 7).

### Historic isolates

In addition to the contemporary field isolates obtained in the study, 53 Australian isolates of *B*. *hyodysenteriae* collected in the 1980s through the early 2000s were obtained from the culture collection at the Reference Centre for Intestinal Spirochaetes at Murdoch University, based on their availability. All had been recovered from diagnostic samples submitted from herds with suspected SD. The isolates comprised 21 from the 1980s, 13 from the 1990s, and 19 from the first decade of the 2000s. They had previously been typed by MLST, and in this study they were subjected to the same analyses for the plasmid-encoded genes as were the isolates from 2014/16.

### Culture and PCR

The diagnostic samples were swabbed onto selective Trypticase Soy agar (BBL) plates (TSA) containing 5% (vol/vol) defibrinated ovine blood, 400 μg of spectinomycin per ml, and 25 μg each of colistin and vancomycin (Sigma–Aldrich) per ml, and were cultured for 5 to 7 days at 37°C in a culture jar with an anaerobic atmosphere generated by an AnaeroGen™ 2.5L Sachet (Oxoid). Zones of haemolysis around the inoculated area indicated growth, and confirmation was obtained that spirochaetes were present by resuspending surface growth in phosphate buffered saline (PBS) and viewing with a phase-contrast microscope. Samples that contained spirochetes were tested concurrently by previously described polymerase chain reactions (PCRs) for *B*. *hyodysenteriae*, *Brachyspira pilosicoli* [16], *B*. *hampsonii* [4], *Brachyspira intermedia* and a genus-specific PCR for *Brachyspira* spp. [17], as well as by a *nox*-RFLP assay for *B*. *suanatina* [18]. New PCR tests for *B*. *hampsonii* and *B*. *suanatina* were developed for use in this study incorporating unique primers targeting the hemolysin A gene (*tlyA*) and DNA-dependent RNA polymerase gene (*rpoC*), respectively. The primers for the new *B*. *hampsonii* PCR were Bham-tlyA-F26 (5’ GTAAAGGCTATACAGAAAGCAGATC 3’) and Bham-tlyA-R376 (5’ CTATGGATATTACTCTATTATCATTA 3’), which anneal to position 26 to 50 and position 351 to 376 of the *tlyA* gene, respectively, giving a product of 351 bp. The primers for the *B*. *suanatina* PCR were Bsua-rpoC-F409 (5’ GATCCAGGCGATAGTTCTTATAC 3’) and Bsua-rpoC-R749 (5’ GTCGCAAATCTTCCGCCGTC 3’), and these anneal to the RNA polymerase gene (*rpoC*) at position 409 to 431 and position 730 to 749, respectively. The PCR product was 341 bp. The specificity of all the PCR assays were confirmed using genomic DNA from 282 *Brachyspira* strains from seven species or proposed species, obtained from the culture collection at the Reference Centre for Intestinal Spirochaetes at Murdoch University. These included strains of *B*. *hyodysenteriae* (n = 81), *B*. *pilosicoli* (n = 107), *B*. *intermedia* (n = 48), *B*. *innocens* (n = 22), *B*. *murdochii* (n = 20), *B*. *hampsonii* (n = 3) and *B*. *suanatina* (n = 2). PCR assays were applied to growth harvested from the primary isolation plates and resuspended in sterile water. The PCR assays were performed in 25 μl reactions consisting of 1x PCR buffer, 1.5 mM MgCl_2_, 0.5 U *Taq* DNA polymerase, 0.2 mM of each dNTP and 0.5 μM of forward and reverse primers. Cycling conditions involved an initial denaturation at 94°C for 5 min, followed by 30 cycles of denaturation at 94°C for 30 s, annealing at 58°C for 15 s and primer extension at 72°C for 1 min. The products were separated by gel electrophoresis and visualised over UV light after staining with ethidium bromide.

### Multilocus sequence typing (MLST) of *B*. *hyodysenteriae* isolates

Ninety-six *B*. *hyodysenteriae* isolates obtained in 2014/16 in the current study were typed by MLST, and MLST data for the other 53 Australian *B*. *hyodysenteriae* isolates that had been recovered from the 1980s to the early 2000s were downloaded from the PubMLST site (http://pubmlst.org/brachyspira/). The isolates were cultured on fresh TSA plates at 37°C for 5 days, harvested, counted and resuspended with sterile PBS as described above. High molecular weight DNA was extracted using the DNeasy Blood and Tissue Kit (Qiagen) according to the manufacturer’s instructions. Cells from a 10 ml sample of a 10^8^ cells per ml culture of *Brachyspira* were harvested by centrifugation at 5,000 *g*. The cell pellet was resuspended in 180 μl of lysis buffer containing 20 μl of proteinase K (10 mg/ml) and incubated at 55°C for 30 min. After lysis, 180 μl of AL Buffer was added and the sample incubated at 70°C for 10 min. Two hundred μl of absolute ethanol was immediately added to the sample and this was transferred to a DNeasy column. Column wash buffers AW1 and AW2 were added sequentially to the columns and centrifuged at 6,000 *g*. The flow-through was discarded, and the DNA was eluted with elution buffer and stored at -20°C.

MLST was conducted as previously described [19]. PCR assays were performed in 50 μl reactions consisting of 1x PCR buffer, 1.5 mM MgSO_4_, 0.5 U HotStar HiFidelity DNA polymerase (Qiagen), 0.2 mM of each dNTP and 0.5 μM of forward and reverse primers. Cycling conditions involved an initial enzyme activation step at 95°C for 15 min, followed by 35 cycles of denaturation at 94°C for 30 s, annealing at 50°C for 15 s and primer extension at 72°C for 1 min. The products were separated by gel electrophoresis and visualised after staining with ethidium bromide. PCR products used for sequencing were purified with the UltraClean™ PCR Clean-up Kit (Mo Bio Laboratories) according to the manufacturer’s instructions. The purified PCR products were used for sequencing, with reactions performed in 10 μl volumes using the BigDye® Terminator v3.1 Cycle Sequencing Kit (Life Technologies) according to the manufacturer’s instructions. An annealing temperature of 50°C was used. The sequencing products were analysed using the Applied Biosystems 3730XL DNA analyser.

The raw sequences were edited and analysed using Geneious version 7.1.7 (Biomatters Ltd). For each locus the consensus sequences were aligned with the *B*. *hyodysenteriae* strain WA1 sequence downloaded from the PubMLST database (www.pubmlst.org) and the aligned loci sequences were trimmed for subsequent MLST analysis, as previously reported [19]. Allele designations for each locus were obtained by a query search from the PubMLST website. New sequence types (STs) were deposited at the PubMLST site.

MLST dendograms were constructed from the data matrix of allelic mismatches using the UPGMA (unweighted-pair group method with allelic arithmetic means) method with 1000 bootstrap replicates using the START2 program [20], and Minimum Spanning Trees (MST) were constructed using the PHYLOViZ software [21]. Consensus sequences were used for the MLST from combined individual distance matrices of nucleotide sequences from seven genes *adh*, *alp*, *est*, *gdh*, *glpK*, *pgm* and *thi*. Clonal complexes (Cc) amongst the isolates were identified by the BURST alogorithm using the eBURST v3 program [22]. Color-coding of STs in the various MSTs was added to distinguish reported health status of the 2014/16 isolates, to compare plasmid gene content, to highlight the decade of isolation, or to show the relationships of isolates from different countries.

### Testing *B*. *hyodysenteriae* isolates for plasmid genes

High molecular weight DNA was extracted from the 96 *B*. *hyodysenteriae* isolates from the field study and 53 historical isolates, as described above. PCR tests were undertaken targeting members of a block of six genes on the 36 kb plasmid of *B*. *hyodysenteriae* [23] that recently have been described as possible virulence-associated genes involved in colonization by *B*. *hyodysenteriae* [24]. These have been designated as open reading frames (orfs) 11, 12, 13, 14, 15 and 16, respectively. For each orf, three primer pairs were designed for PCR amplification, as previously described [24]. Two PCRs also were conducted to detect the presence of the whole 36 kb plasmid, as previously described [24]. PCR assays were performed in 25 μl reactions, as described previously, with the exception that the annealing temperature used for each primer was set at 5°C less than the optimal annealing temperature to allow for a moderate stringency reaction. Amplification products were electrophoresed through an agarose gel, stained with ethidium bromide and viewed over ultraviolet light.

Each of the six orfs was recorded as being either absent (N) or present (P), and the combination was used to define a “plasmid type”, with numbers designated 1 through 7: 1 = N, N, N, N, N, N; 2 = N, N, N, N, N, P; 3 = N, N, P, N, P, P; 4 = N, N, P, P, P, P; 5 = N, P, P, N, P, P; 6 = P, P, P, N, P, P; and 7 = P, P, P, P, P, P. Assuming no other genomic differences, an isolate with a plasmid type 1, without any of the block of plasmid genes, would be predicted to be less capable of colonizing than one with a plasmid type 7, having all six of the genes. Statistical comparisons between the distribution of isolates with plasmid types 1 and 7 were made using Fisher’s exact test in GraphPad Instat. Pairwise comparisons were made between the three reported herd health statuses for the 2014/16 isolates, and between the isolates from the four decades originating from the 1980s to 2014/16. Comparisons for other plasmid types were not made because the relative significance of the presence/absence of individual genes in the block remains unclear, and there were too few occurrences of these intermediate scores to make statistical analysis meaningful.

### Antimicrobial susceptibility testing

The *B*. *hyodysenteriae* isolates to be tested for antimicrobial susceptibility were subcultured to ensure purity and then grown on fresh TSA plates at 37°C for 5 days, as described above. The cells were harvested from the agar plate by resuspending the surface growth with 2 ml sterile PBS, transferring this into a microfuge tube and centrifuging the tube at 5,000 *g*. The pellet was resuspended in sterile PBS, the cells counted using a hemocytometer and then diluted to a density of 10^6^ cells per ml.

Antimicrobial susceptibility of the isolates from 2014/16 was assessed using the agar-dilution method. The test plates consisted of TSA containing 5% defibrinated ovine blood and the appropriate antibiotic concentration. Control plates did not include antibiotics. The plates were incubated for 5days at 37°C in anaerobic jars and then observed for hemolysis. The isolates were tested for susceptibility to varying concentrations of lincomycin (2, 4, 16, 36, and 72 μg/ml), tylosin (1, 5, 25, 50 and 100 μg/ml) and tiamulin (0.25, 0.5, 1, 4 and 8 μg/ml). These antimicrobials and dilutions were selected to replicate earlier studies to allow comparisons of results to be made over time.

A total of 10^5^ cells were drop-inoculated onto the control and sensitivity plates. Each isolate was tested in duplicate and *B*. *hyodysenteriae* control strain WA1 was included in each batch of tests. Growth of the strains on the control and sensitivity plates was checked visually after 5 days incubation. Zones of hemolysis present around the growth on the control plates were determined, and isolates were recorded as being susceptible to the antimicrobial concentration in the test plates if no such zones were observed. Surface growth was scraped off the plate and examined under a phase contrast microscope to confirm purity and the endpoint. The first sensitive colony zone and the last resistant colonies were checked for spirochete growth by phase-contrast microscopy. The minimum inhibiting concentration (MIC) of the antimicrobial was reported as the lowest concentration of that inhibited growth. MIC breakpoints used to assist interpretation of the results are presented in Table 1. Results were compared with those reported for Australian isolates for 2002–2006 and 2006–2007 that previously had been tested using the same methodology [25].

**Table 1:** MIC breakpoints (μg/ml) for *in-vitro* antimicrobial susceptibility tests performed on *Brachyspira hyodysenteriae* isolates

Antimicrobial	Sensitive	Intermediate	Resistant	Reference
Lincomycin	≤ 4	> 4 ≤ 36	> 36	[26]
Tylosin	≤ 1	> 1 ≤ 4	> 4	[26]
Tiamulin	≤ 0.25	> 0.25 ≤ 2	> 2	[27]

## Results

### PCR assay results

The established and the two newly developed PCRs targeting the new species were shown to amplify the gene from the target species when tested on the collection of 282 *Brachyspira* species and strains, and did not show any cross-reactivity. The new PCR described here for *B*. *hampsonii* gave stronger bands with members of this species than did the previously described PCR for this species, whilst the PCR for *B*. *suanatina* was quicker and easier to perform than the *nox*-RFLP assay.

### Detection of *Brachyspira* species

Neither *B*. *hampsonii* nor *B*. *suanatina* were detected in any of the samples tested from the 97 herds, using culture and the previously described and the newly developed PCR methods. *B*. *hyodysenteriae* was detected in 159 samples (14.4%) from 34 herds (35.1%), and 96 isolates successfully were obtained in pure culture from 30 of these herds (Table 2). In the cases where *B*. *hyodysenteriae* isolates were unable to be recovered for further study from positive plates, this was due to multiple *Brachyspira* species being present and/or overgrowth with contaminant microorganisms. Two *B*. *hyodysenteriae* isolates, one each from herd 28 (ST151) and herd 86 (ST161) respectively, were weakly beta-hemolytic, and both herds were considered to be free of SD. Three unconnected herds that were colonized with *B*. *hyodysenteriae* supplied stock to other production herds. Of the original 34 herds identified as being colonized by *B*. *hyodysenteriae*, 14 of 24 (58.3%) were identified through heightened abattoir surveillance of apparently healthy herds, 12 of 25 (48%) were already suspected to have SD based on clinical signs or past history, and eight of 48 (33.3%) were of uncertain health status. Isolates were not obtained in pure culture from one of the herds that was identified as infected through abattoir surveillance, one considered to have SD, and two which were originally of uncertain health status.

**Table 2:** Comparison of sequence type (ST), plasmid gene profile (PT) and antimicrobial susceptibility profiles of 96 *B*. *hyodysenteriae* isolates recovered in 2014/16 belonging to 30 STs present in 30 herds in different Australian States that either were thought to be free of swine dysentery (SD), to have the disease, or to be of uncertain health status

Herds thought to be free of SD (13 herds, 12 STs, 39 isolates)			Sequence type (ST), plasmid gene type (PT) and antimicrobial susceptibility profiles^a^				
Australian State^b^	Herd code	No of isolates with same profile^c^	ST^d^	PT	Lincomycin MIC	Tylosin MIC	Tiamulin MIC
NSW	Herd 5	2	160	1	R (≥36>72)	R (≥100)	I (<0.5)
NSW	Herd 5	5	150	7	R (≥36>72)	R (≥100)	S (<0.25)
SA	Herd 20	2	147	1	R (≥72)	R (≥100)	I (≥1<2)
SA	Herd 20	1	148	1	R (≥72)	R (≥5<25)	I (<0.5)
SA	Herd 27	3	49	1	R (≥72)	R (≥100)	I (≥1<2)
SA	Herd 28	1	151	1	R (≥72)	R (≥100)	I (<0.5)
VIC	Herd 50	2	150	7	S (<2)	I (≥1<5)	I (<0.5)
NSW	Herd 62	2	150	7	R (≥72)	R (≥100)	I (<0.5)
VIC	Herd 63	1	50	1	R (≥36<72)	R (≥100)	I (<0.5)
VIC	Herd 63	1	50	1	I (≥16<36)	R (≥100)	I (≥0.5<1)
VIC	Herd 63	2	153	7	I (≥4<16)	R (≥5<25)	I (<0.5)
VIC	Herd 68	2	150	7	R (≥36>72)	R (≥5<25)	I (<0.5)
VIC	Herd 70	1	150	7	S (<2)	I (≥1<5)	I (<0.5)
VIC	Herd 80	1	159	1	R (≥72)	R (≥100)	R (≥4<8)
WA	Herd 86	3	50	1	S (<2)	R (≥25<50)	I (≥0.5<1)
WA	Herd 86	1	161	1	S (<2)	R (≥5<25)	I (<0.5)
WA	Herd 86	1	50	1	S (<2)	I (≥1<5)	S (<0.25)
WA	Herd 86	1	50	1	R (≥72)	R (≥100)	S (<0.25)
WA	Herd 86	1	50	1	R (≥72)	I (≥1<5)	S (<0.25)
WA	Herd 87	4	140	4	S (<2)	R (≥100)	I (<0.5)
WA	Herd 88	2	144	1	R (≥72)	R (≥100)	S (<0.25)
**Herds reported to have SD (11 herds, 17 STs, 35 isolates)**							
NSW	Herd 2	1	157	1	I (≥16<36)	R (≥100)	I (<0.5)
NSW	Herd 2	2	162	1	S (<2)	R (≥5<25)	S (<0.25)
NSW	Herd 2	1	140	4	R (≥72)	R (≥100)	I (<0.5)
NSW	Herd 2	3	143	4	R (≥72)	R (≥100)	I (<0.5)
NSW	Herd 3	1	145	1	S (<2)	R (≥5<25)	I (<0.5)
NSW	Herd 3	1	145	1	I (≥4<16)	R (≥50<100)	S (<0.25)
NSW	Herd 3	1	146	1	NT^e^	NT	NT
NSW	Herd 3	1	154	1	I (≥4<16)	R (≥50<100)	S (<0.25)
SA	Herd 21	2	49	1	R (≥72)	R (≥5<25)	I (<0.5)
SA	Herd 37	1	152	1	R (≥72)	R (≥100)	I (<0.5)
VIC	Herd 46	2	150	7	R (≥72)	R (≥100)	R (≥8)
VIC	Herd 49	1	158	2	R (≥36>72)	R (≥100)	R (≥4<8)
VIC	Herd 71	4	166	3	R (≥72)	R (≥100)	R (≥8)
VIC	Herd 71	1	166	4	R (≥72)	R (≥100)	R (≥8)
VIC	Herd 73	1	155	1	I (≥4<16)	R (≥100)	I (<0.5)
VIC	Herd 73	1	156	1	I (≥16<36)	R (≥100)	I (<0.5)
VIC	Herd 73	8	144	1	R (≥72)	R (≥100)	S (<0.25)
VIC	Herd 75	1	149	1	R (≥72)	R (≥100)	I (<0.5)
VIC	Herd 78	1	163	4	I (≥4<16)	R (≥5<25)	S (<0.25)
VIC	Herd 85	1	143	4	R (≥72)	R (≥100)	S (<0.25)
**Herds of uncertain disease status (6 herds, 8 STs, 22 isolates)**							
QLD	Herd 8	4	165	1	R (≥72)	R (≥100)	I (<0.5)
SA	Herd 12	1	164	3	R (≥72)	R (≥100)	I (<0.5)
QLD	Herd 13	1	141	1	NT	NT	NT
QLD	Herd 13	1	142	1	NT	NT	NT
VIC	Herd 47	5	150	7	R (≥72)	R (≥5<25)	R (≥8)
WA	Herd 95	7	31	5	S (<2)	R (≥100)	S (<0.25)
WA	Herd 98	1	144	1	I (≥4<16)	R (≥100)	I (<0.5)
WA	Herd 98	2	140	4	R (≥72)	R (≥100)	I (<0.5)

^a^ST, sequence type in MLST; PT, plasmid gene profile type (see Materials and Methods for definition); S, sensitive; I, intermediate; R, resistant.

^b^QLD, Queensland; NSW, New South Wales; SA, South Australia; VIC, Victoria; WA, Western Australia

^c^Two isolates respectively from herd 28 (ST151) and herd 86 (ST161) were weakly hemolytic

^d^STs 140, 144 and 150 were recovered from pigs from all three categories of health status, and STs 49 and 143 were found in two categories. The other STs were only found in one health status category.

^e^NT, Not tested.

In addition to *B*. *hyodysenteriae*, the potential pathogen *B*. *pilosicoli* was identified in 127 samples (11.5%) from 39 (40.2%) herds; and *B*. *intermedia* in 98 (8.5%) samples from 37 (38.1%) herds. There were 27 (2.4%) samples that were positive for *Brachyspira* spp. but negative for the species-specific PCRs from 17 (17.5%) herds, and these were presumed to contain *B*. *innocens* or *B*. *murdochii*. Sixty-seven individual samples collected from 28 herds contained more than one of the above named *Brachyspira* species, with 57 samples containing two species and ten samples containing all three species.

### Multilocus sequence typing of *B*. *hyodysenteriae*

The 96 *B*. *hyodysenteriae* isolates from 2014/16 belonged to 30 sequence types (STs) (Table 2). Eleven STs contained multiple isolates, and seven of these were identified in more than one herd. The 39 isolates from 13 herds originally considered not to have SD were divided into 12 STs; of these, ST50 was found in two herds and ST150 in five of the 13 herds. One of the latter five was a breeding herd, and the other four herds with this ST are likely to have received pigs from it. This ST also was found in one herd with SD and one of the herds of uncertain health status, making it the most common ST found. The 35 isolates from the 11 herds that originally were believed to have SD belonged to 17 STs, whilst the 22 isolates from the six herds of uncertain SD-status belonged to 8 STs. STs 140, 144 and 150 were found in pigs from all three categories of health status, whilst STs 49 and 143 each were found in two health categories. The other STs each were only found in herds of a single health category. STs 31, 49 and 50 previously have been described in Australian herds sampled in earlier decades, and these were represented amongst the older isolates included in this study. The historic isolate and contemporary isolate in ST31 both came from Western Australia (WA); those in ST49 all came from herds in SA; and those in ST50 came from Queensland, Victoria and Western Australia.

Multiple isolates (2–10) were available for analysis from 20 of the herds, and nine of these herds had more than one ST identified. Of these 20 herds, 10 were reported not to have SD, and four of these had more than one ST identified; five of these herds were reported to have SD, and multiple different STs were found in three of these, with herd 2 having 4 STs and herd 3 having 3 STs; and five of the herds were of unknown health status, of which two had multiple STs.

The 21 isolates from the 1980s were divided into 17 STs; the 13 isolates from the 1990s into 12 STs; and the 19 isolates from the early 2000s into 10 STs (Table 3). As mentioned above, ST31 (1980s), ST50 (1990s) and ST49 (2000s) also were represented amongst the STs from 2014/16 (Table 2).

**Table 3:** Australian State of origin, sequence type (ST) and plasmid profile (PT) of 53 *B*. *hyodysenteriae* isolates belonging to 39 STs, isolated in the 1980s, 1990s and early 2000s and used for comparative purposes

State^a^	Decade	No. of isolates with the same ST^b^ and PT^c^ profile	ST^b^	PT^c^
VIC	1980s	2	12	1
SA	1980s	1	16	1
VIC	1980s	1	17	1
VIC	1980s	1	20	7
QLD	1980s	2	22	7
SA	1980s	1	23	7
SA	1980s	1	25	7
VIC	1980s	1	27	4
VIC	1980s	1	29	4
WA	1980s	1	**31**	7
QLD	1980s	1	32	4
VIC	1980s	1	32	4
WA	1980s	1	36	4
WA	1980s	1	37	7
WA	1980s	1	40	4
WA	1980s	1	43	6
WA	1980s	2	44	4
WA	1980s	1	47	7
VIC	1990s	1	11	4
VIC	1990s	1	14	4
NSW	1990s	2	15	4
NSW	1990s	1	18	3
VIC	1990s	1	20	4
NSW	1990s	1	28	7
NSW	1990s	1	33	7
NSW	1990s	1	34	7
QLD	1990s	1	35	7
QLD	1990s	1	36	7
QLD	1990s	1	47	7
QLD	1990s	1	**50**	7
WA	2000s	1	1	2
WA	2000s	2	2	7
SA	2000s	1	3	7
NSW	2000s	5	19	1
NSW	2000s	2	21	1
SA	2000s	1	**49**	7
WA	2000s	1	61	1
WA	2000s	1	62	1
WA	2000s	1	63	1
WA	2000s	4	64	1

^a^ VIC, Victoria; SA, South Australia; QLD, Queensland; WA, Western Australia; NSW

New South Wales.

^b^ST, sequence type in MLST. STs 31, 49 and 50 marked in bold also were identified amongst the isolates from 2014/16.

^c^PT, plasmid gene profile type (see text and Table 4 for definition). All isolates possessed the plasmid, even where they lacked the block of six genes making up the profile.

**Fig 1:**
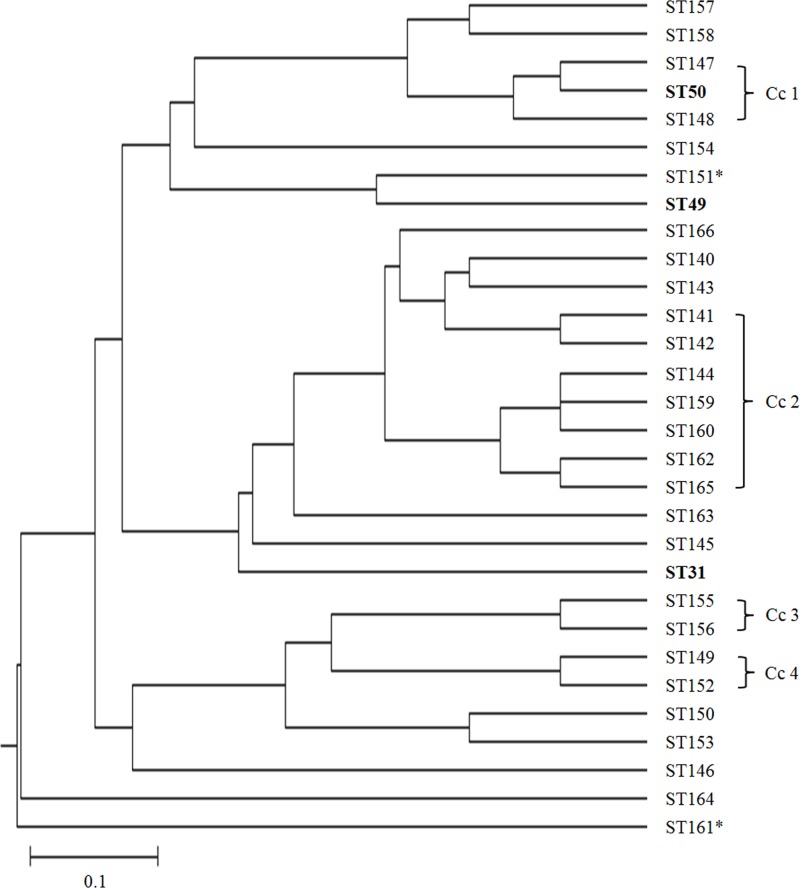
MSLT dendogram showing the 30 STs of the 96 *B*. *hyodysenteriae* isolates from 2014/16. The dendrogram is based on consensus sequences constructed from combined individual distance matrices of nucleotide sequences from seven genes *adh*, *alp*, *est*, *gdh*, *glpK*, *pgm* and *thi*. STs shared with historic isolates found in the PubMLST database are indicated in bold (these were all of Australian origin). The STs of the two weakly hemolytic *B*. *hyodysenteriae* isolates are indicated with an asterisk. The length of the scale bar represents 10-nucleotide substitution in 100 base pairs of the sequenced gene fragment.

An MLST dendrogram demonstrating the relationships of the various STs for isolates for the period 2014/16 is shown as Fig 1. Four clonal complexes were identified by the eBURST program and are marked on the tree. Most isolates were relatively closely related, although ST161 and to a lesser extent ST154 were outliers. The former was weakly hemolytic, although the second weakly hemolytic isolate in ST151 was in the main body of the tree and was not closely related to ST161.

**Fig 2:**
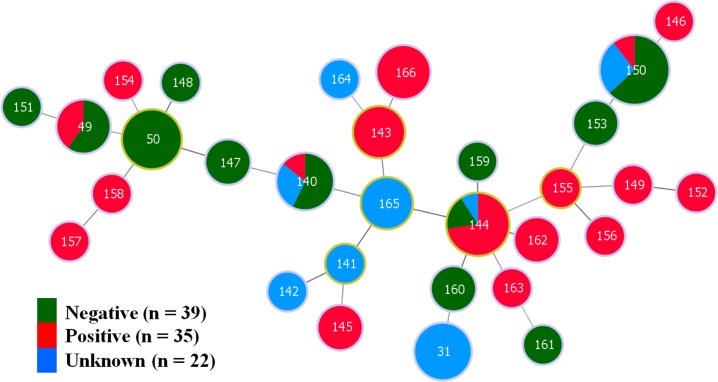
Minimum Spanning Tree (MST) showing relationships of the 96 *B*. *hyodysenteriae* isolates from 2014/16 and the three categories of herd health status that were originally reported. In the MST, each labeled node represents a different ST and the color represents the original reported herd health status (negative, positive or of uncertain health status). The size of the node indicates the number of strains having the same ST. Nodes with a yellow margin show the STs that are founder members of clonal complexes. The STs of the weakly hemolytic isolates identified in this study are indicated with black arrows.

A minimum spanning tree (MST) showing the relationship of the 2014/16 isolates of *B*. *hyodysenteriae* belonging to 30 different STs and the reported disease status of their herds of origin is shown as Fig 2. The STs containing isolates from the three disease status categories were dispersed across the tree, with no clear and consistent clustering of STs related to the health status. Four STs (49, 140, 144 and 150) included multiple isolates from herds with different reported health categories.

**Fig 3:**
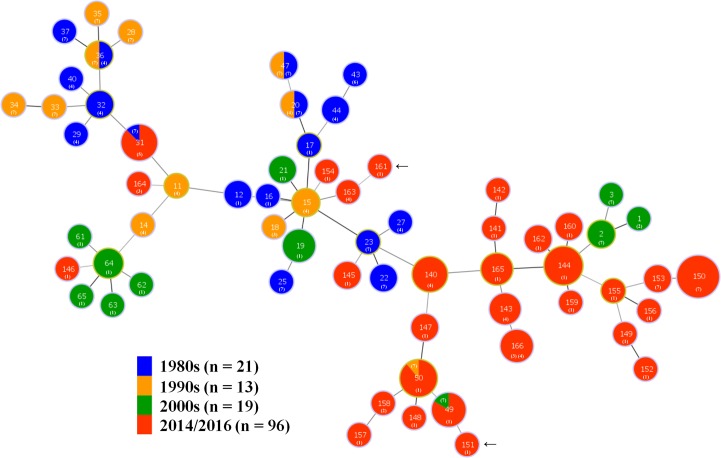
Minimum Spanning Tree (MST) showing relationships of the 96 *B*. *hyodysenteriae* isolates from 2014/16 and 53 previously described Australian isolates whose data was obtained from PubMLST. In the MST, each labelled node represents a different ST and the color represents the decade of origin. The size of the node indicates the number of strains having the same ST. Nodes with a yellow margin show the STs that are founder members of clonal complexes. The plasmid types of the isolates in the STs are shown with a numeral, labelled 1 to 7 as defined in Table 4. The STs of the weakly hemolytic isolates identified in this study are indicated with black arrows.

An MST showing the relationship of the STs for the 2014/16 isolates and the STs of the 53 historic Australia isolates from the 1980s to early 2000s is presented as Fig 3. Most of the STs containing isolates from 2014/16 were located in clusters placed away from the STs containing isolates from previous decades, although there was a tendency for STs containing isolates from consecutive decades to be more closely related to each other than to those from other decades. Six STs contained multiple isolates collected in different decades, and of these STs 31 and 50 contained isolates from the 2014/16, as well as from the 1980s or 1990s, respectively. The plasmid type of the isolates also is shown.

**Fig 4:**
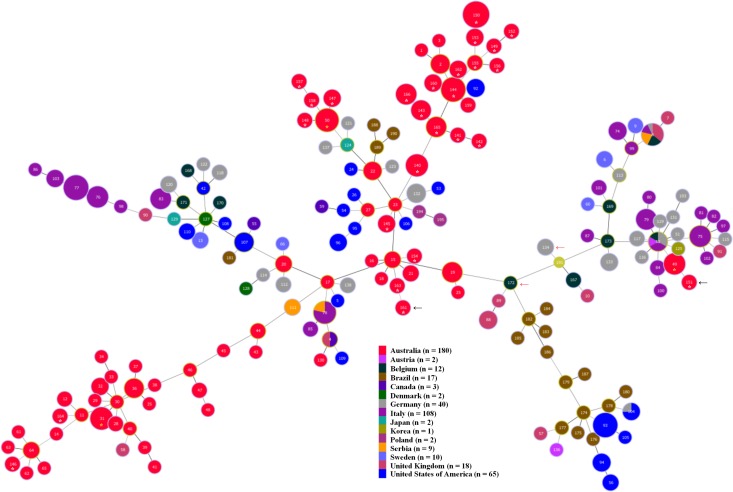
Minimum Spanning Tree showing relationships of the 96 Australian *B*. *hyodysenteriae* isolates from 2014/16 in this study and 375 previously described isolates, data for which was obtained from PubMLST. The isolates that are shown originated from Australia, Austria, Belgium, Brazil, Canada, Denmark, Germany, Italy, Japan, Korea, Poland, Serbia, Sweden, the United Kingdom and the United States of America. In the MST, each labelled node represents a different ST and the color represents the country of origin. The size of the node indicates the number of strains having the same ST. The STs identified from the current study are indicated with an asterisk. Newly recognised STs are indicated with a white asterisk and previously described STs are indicated with a yellow asterisk. The positions of the weakly hemolytic isolates identified in this study are indicated with a black arrow. A red arrow indicates the position of previously described weakly hemolytic *B*. *hyodysenteriae* isolates from Germany [14] and Belgium [28].

A comparison of all the STs containing Australian isolates with those for isolates from other countries by MST is presented as Fig 4. The majority of the STs containing the Australian isolates were in broad clusters that were generally arrayed apart from the STs containing isolates from other countries. None of the STs containing Australian isolates were shared with previously described STs from other countries.

### Virulence-associated plasmid genes

The distribution of members of the block of six plasmid genes in STs of *B*. *hyodysenteriae* isolates from 2014/16 is shown in Table 4 for each of the three health statuses that were originally reported. The distribution of STs with different numbers of the block of six plasmid genes was not obviously different across the three reported health statuses. Between half and three quarters of the STs in the three categories had plasmid profile 1, lacking all six genes, whilst few STs had isolates with all six of the block of plasmid genes. In Fisher’s exact test no significant differences were found between the distribution of isolates with plasmid types 1 and 7 in the three reported health statuses. Three isolates, one each from herd 73 (ST155), herd 28 (ST151), and herd 86 (ST161) respectively lacked the entire plasmid. The latter two isolates also were weakly hemolytic. Herds 5, 63, 2, 71 and 98 contained isolates with different STs that had different plasmid types (eg types 1 and 7 both were found in isolates from herds 5 and 63).

**Table 4:** Number and percentage of the 30 STs of *B*. *hyodysenteriae* isolates from the 2014/16 period possessing different combinations of the six plasmid-borne virulence-associated genes (plasmid types)

Plasmid type^b^	Number and percentage of STs^a^ with different plasmid types		
	Amongst 13 herds thought to be uninfected	Amongst 11 herds reported to have SD	Amongst 6 herds of uncertain health status
1 (N, N, N, N, N, N)	9 (75%)	11 (64.7%)	4 (50%)
2 (N, N, N, N, N, P)	-	1 (5.9%)	-
3 (N, N, P, N, P, P)	-	-	1 (12.5%)
4 (N, N, P, P, P, P)	1 (8.3%)	4 (23.5%)	1 (12.5%)
5 (N, P, P, N, P, P)	-	-	1 (12.5%)
6 (P, P, P, N, P, P)	-	-	-
7 (P, P, P, P, P, P)	2 (16.7%)	1 (5.9%)	1 (12.5%)
Total number of STs	12	17	8

^a^ST, sequence type in MLST (total of 30 STs identified). Three STs (140, 144 and 150) were common to all three of the health status categories, and two ST (49 and 143) each were common to two categories.

^b^Plasmid types defined by the absence (N) or presence (P) of the six plasmid genes in numerical order (orf11, orf12, orf13, orf14, orf15, orf16).

The distribution of STs with respect to members of the block of six plasmid genes in *B*. *hyodysenteriae* isolates from the 1980s, 1990s, early 2000s and 2014/16 is summarized in Table 5, and also shown in Fig 3. Apart from three isolates recovered in 2014/16, all possessed the 36 kb plasmid. A greater percentage of isolates from the 1980s and 1990s possessed all six plasmid genes (plasmid type 7) than did isolates in the early 2000s and in 2014/16, where plasmid type 1 (none of the genes present) was more common. Results of the statistical comparison between the distributions of isolates with these two plasmid types over these decades are presented in Table 6. The isolates from 2014/16 had highly significantly more isolates with plasmid type 1 compared to type 7 than did those for the 1980s and for the 1990s. The isolates from the 2000s had significantly more isolates with type 1 compared to type 7 than did the isolates from 1990s. No other differences were significant. Three herds (herds 2, 3 and 63) sampled in 2014/16 had multiple isolates that belonged to different STs with very different plasmid gene types (type 1 in all three herds, together with isolates of either type 7 or 4). Comparison of the three STs that included isolates both from 2014/16 and previous decades showed that the isolate in ST31 from the 1980s had plasmid type 7, as did the isolates in 2014/16; the isolate in ST50 from the 1990s had plasmid type 7 whilst the isolates from 2014/16 had plasmid type 1; and the isolate in ST49 from the early 2000s had plasmid type 7 whilst the isolates from 2014/16 had plasmid type 1. Again this is consistent with a tendency for isolates to have progressively lost plasmid genes since the 1980s.

**Table 5:** Number and percentage of STs of recent and historical *B*. *hyodysenteriae* isolates possessing different combinations of the six plasmid-borne virulence-associated genes

Plasmid type^a^	Number and percentage of STs^b^ with different plasmid types			
	1980s	1990s	2000s	2014/16
1 (N, N, N, N, N, N)	3 (17.6%)	- (0%)	6 (60%)	21 (70%)
2 (N, N, N, N, N, P)	-	-	1 (10%)	1 (3.3%)
3 (N, N, P, N, P, P)	-	1 (8.3%)	-	1 (3.3%)
4 (N, N, P, P, P, P)	6 (35.3%)	4 (33.3%)	-	4 (13.3%)
5 (N, P, P, N, P, P)	-	-	-	1 (3.3%)
6 (P, P, P, N, P, P)	1 (5.9%)	-	-	-
7 (P, P, P, P, P, P)	7 (41.2%)	7 (58.3%)	3 (30%)	2 (6.7%)
Total no. of STs	17	12	10	30

^a^ Plasmid types defined by the absence (N) or presence (P) of the six plasmid genes in numerical order (orf11, orf12, orf13, orf14, orf15, orf16).

^b^ST, sequence type in MLST (total of 69 STs; duplicate STs for 2014/16 removed).

**Table 6:** Matrix showing two-tailed P values of significance of differences in distribution of plasmid types 1 and 7 between isolates from the four decades using Fisher’s exact test

Decade	1980s	1990s	2000s
**1980s**	-	-	-
**1990s**	0.2279	-	-
**2000s**	0.1789	0.0114	-
**2014/16**	0.0008	< 0.0001	0.1206

### Antimicrobial susceptibility of *B*. *hyodysenteriae* isolates

The antimicrobial susceptibility status to lincomycin, tylosin and tiamulin for isolates in STs of *B*. *hyodysenteriae* isolated in 2014/16 is shown in Table 2, and a summary comparing results for 2014/16 with previously reported results for Australian isolates from 2002–2006 and 2006–2007 [25] is presented in Table 7. Most or all isolates from all three periods were resistant to tylosin. Approximately 61% of isolates from 2014/16 were resistant to lincomycin, and this figure was considerably increased compared the results from both earlier periods. Tiamulin resistance in 2014/16 was more common (15.2%) than amongst the isolates from 2006–2007, and there was a high percentage (58.7%) of isolates of intermediate susceptibility in 2014/16. The distribution of susceptibility for tiamulin for the isolates from 2002–2006 was more similar to the data for the 2014/16 isolates than for the 2006–2007 isolates.

**Table 7:** Classification of the *B*. *hyodysenteriae* isolates collected in 2014–2016 as being susceptible, intermediate or resistant to the three antimicrobials, and comparison with reported results for Australian isolates from previous periods

Period (no. of isolates)	Antimicrobial	No. (%) susceptible	No. (%) intermediate	No. (%) resistant
2014–2016 (n = 46)^a^	Lincomycin	9 (19.6%)	9 (19.6%)	28 (60.9%)
	Tylosin	-	4 (8.9%)	42 (91.3%)
	Tiamulin	12 (26.1%)	27 (58.7%)	7 (15.2%)
2006–2007 (n = 60)^b^	Lincomycin	19 (31.6%)	31 (51.6%)	10 (16.6%)
	Tylosin	-	-	60 (100.0%)
	Tiamulin^c^	57 (95%)	2 (3.3%)	1 (1.6%)
2002–2006 (n = 89)^b^	Lincomycin	26 (29.2%)	57 (64%)	6 (6.7%)
	Tylosin^d^	-	2 (2.7%)	73 (97.3%)
	Tiamulin^c^	16 (18%)	62 (70%)	11 (12.4%)

^a^Multiple isolates from the same herd with the same overall profile only counted once

^b^Results from Hampson [25]

^c^Results recalibrated according to the criteria of Pringle et al. [27].

^d^Fourteen isolates not tested

Isolates belonging to 4 STs (STs 150, 158, 159 and 166) were not susceptible to all three of the antimicrobials that were tested (ie were multi-drug resistant isolates), and were identified in five Victorian herds, with ST150 being present in two herds. Two of these herds supplied pigs to other herds. Other isolates of ST 150 in five other herds had different patterns of susceptibility to the three antimicrobials. Furthermore, there were examples where multiple isolates of the same ST in a single herd had different susceptibility patterns (ST 50, herd 86), or where isolates of different STs in the same herd had different susceptibility patterns (eg in herds 2 and 86).

## Discussion

It is thought that pigs were introduced into Australia in 1788, arriving on ships of the first fleet from England. Subsequent imports of live pigs from different parts of the world occurred until the mid-1980s, when importation was banned. It has been presumed that carrier pigs introduced SD into Australia on one or more occasions, with subsequent spread throughout the country. In the northern hemisphere, movement of migratory water birds also has been shown to be a potential source of infection, as they may carry strains of *B*. *hyodysenteriae*, *B*. *suanatina* and *B*. *hampsonii* [7,9,11,29,30]: potentially such migratory avian species may be another means by which *Brachyspira* species have been introduced into Australia and/or transmitted between herds.

In the current study neither *B*. *suanatina* nor *B*. *hampsonii* were identified in the 97 herds examined, whether or not they had clinical signs of disease. These findings do not exclude the possibility that these species occur in Australia, but it shows that if they are present they are uncommon, and so are not currently an important issue for the Australian Pig Industry. Although it seems that introduction of these species by migratory water birds is unlikely to have occurred, nevertheless it would be useful to sample such species as they arrive into Australia from Asia to determine whether they carry these and other *Brachyspira* species. Another implication from the findings is that those strains of *B*. *hyodysenteriae* that currently are present in Australia are more likely to have evolved from strains imported with pigs before the mid-1980s, rather than having been introduced by migratory birds since then. Furthermore, the colonization of feral pigs in Australia by *B*. *hyodysenteriae* is likely to have arisen by direct or indirect transfer of strains to them from commercial pigs with SD [31].

Interestingly, members of another novel strongly beta-hemolytic spirochete that was recovered from pigs with an SD-like disease on an Australian piggery in 2007 also were not identified, suggesting that this too is an uncommon species infecting pigs [32]. On the other hand, the potentially pathogenic *B*. *pilosicoli* was identified in 40% of the herds, whilst *B*. *intermedia* was identified in 37%, and other presumed non-pathogenic weakly hemolytic *Brachyspira* spp. in 17.5% of the herds. The latter were not investigated further by sequencing the PCR products, but are likely to have included *B*. *innocens*, *B*. *murdochii* and possibly “*B*. *pulli*” [33]. The prevalence rates for *B*. *pilosicoli* and for *B*. *intermedia* were somewhat higher than those recorded in Danish finisher pigs in herds with diarrhea (*B*. *pilosicoli* 19% of herds; *B*. *intermedia* 13%; *B*. *innocens* 34%) [34]. These results suggest that the methodologies used for isolation and identification of the *Brachyspira* species in this study were appropriate, and show that both *B*. *pilosicoli* and *B*. *intermedia* are widespread amongst Australian pig herds.

An important finding was the identification of *B*. *hyodysenteriae* in diagnostic samples from 14 of 24 (58.3%) herds where the consultant veterinarian/management initially considered that infection was not present. These included breeding herds that underwent heightened surveillance as part of this study, including collection of colonic mucosa from healthy pigs at the abattoir. Results for six of these herds that were initially identified as infected through serological screening have been reported previously [15]. A similar situation was reported in an early Australian study, where an isolate of *B*. *hyodysenteriae* was recovered from a pig in a high-health status herd with minimal antimicrobial usage and no disease [35]. In this case the isolate caused SD in pigs that subsequently were experimentally challenged with it in a research facility. The occurrence of unrecognized colonization of herds by *B*. *hyodysenteriae* is not limited to Australia, as recently colonization has been demonstrated in apparently healthy breeding herds in both Switzerland [36] and Germany [14]. In addition, eight of the 24 herds (33.3%) of uncertain health status in the current study were identified as being colonized with *B*. *hyodysenteriae*. Some of these herds had pigs with mild diarrhea or colitis, but these signs had not been attributed to SD because of their mild nature. Together these findings emphasize that colonization with *B*. *hyodysenteriae* in apparently healthy herds and herds with mild disease not necessarily attributed to SD may be much more common than previously has been realized. On the other hand, in the current study only 12 of 25 (48%) herds that were already suspected to have SD based on clinical signs or past history were successfully identified as being colonized by culture and PCR of feces and/or colonic samples. This raises the possibility that even more of the “SD free” herds or those of uncertain health status might have been colonized than were detected. The results emphasize the importance of all herds routinely and regularly undertaking abattoir checks, including taking colonic mucosal samples and using specific laboratory testing to confirm their colonization status, and particularly so if they supply pigs to other herds.

The MLST method was shown to be very useful for studying the molecular epidemiology of *B*. *hyodysenteriae* in Australia, as has previously been reported [37–39]. Sequence types (STs) were equated to strains, although it was recognized that minor differences can occur between isolates in the same ST. Transfer of isolates with the same STs between herds was seen in the case of ST150 where isolates were present in a breeding herd and in other herds that received pigs from it. The finding of multiple different strains in some herds was significant, and rarely has been reported previously [40]. It is problematic as it could complicate control measures in herds where the isolates have mixed phenotypes, as for example with the different antimicrobial susceptibility patterns found amongst the different STs present in herd 86. Indeed, in this herd different isolates of ST50 also had different antimicrobial susceptibility profiles.

Overall the MLST results show that *B*. *hyodysenteriae* isolates present in Australian herds in 2014/16 were genetically diverse. Importantly, the MST presented as Fig 2 shows that isolates from herds with the three different reported health statuses were not clustered according to this status. Consequently, as previously reported [14], the disease outcome following colonization is not necessarily related to the genetic background of the isolate as reflected by its ST; instead, relative virulence potential is more likely to reside with strain-specific attributes that are not part of the core genome functions.

When comparing the Australian isolates from 20014/16 with those from previous decades, only six STs were shared in different decades (Fig 3) and there was an apparent evolutionary drift of strains over this period. Evidence for micro-evolution of *B*. *hyodysenteriae* strains over short periods of time previously has been obtained on Australian farms by comparing DNA patterns of isolates using pulsed field gel electrophoresis [40]. An accumulation of such genetic drift over time presumably contributes to the current diversity of the isolates in Australia. These changes are overlain with transmission of strains between herds through infected pigs and by indirect routes such as by movement of trucks, personnel or potential reservoir species such as feral pigs, rodents, dogs and birds. The STs containing Australian isolates all were distinct from those so far identified in other countries (Fig 4). This and the diversity found emphasizes that the current Australian strains are most likely to have originated from a number of importations of infected pigs, and that these strains have evolved considerably and independently since they were introduced.

An important practical question that arises from the study is why some herds that were colonization, including breeding herds, apparently did not have clinical signs of SD. In some cases antimicrobial use might be controlling and masking disease, although none of the herds in this study reported this as being a potential contributing factor, and it is unlikely to apply in all cases. Diet is another potential explanation, as different ingredients have been reported to influence the occurrence and severity of SD in infected pigs and herds, presumably by altering the physicochemical environment and the composition of the microbiota in the large intestine [41–48]. In this case, should isolates from healthy herds be transmitted to other herds where different diets are used then disease eventually may develop [13]. In the current study, however, the majority of herds that were tested were fed standard Australian commercial diets mainly based on wheat and plant protein, so it seems unlikely that diet is an important common contributor to the current observations.

Another possible explanation for the apparent lack of disease is that the isolates of *B*. *hyodysenteriae* recovered from healthy herds were avirulent or had reduced virulence potential, as has been described previously for some isolates in Europe and North America [49–52]. Hemolytic activity is believed to be an important virulence factor in *B*. *hyodysenteriae* [1], and genetically distinct isolates from two of the herds initially believed to be free of SD were weakly beta-hemolytic. This phenotype also has been reported in isolates from European pigs [28], including in an apparently avirulent isolate recovered from a healthy German breeding herd [14]. Although this finding may help to explain lack of disease in these two herds, in herd 86 another ST containing strongly hemolytic *B*. *hyodysenteriae* isolates also was found with the weakly hemolytic isolate. In this case the isolates from the other ST also might be predicted to have reduced virulence potential for other reasons, as no disease was observed.

Besides hemolysis, the block of six plasmid genes may encode other potential virulence factors that have been loosely associated with facilitating colonization [15,24,53]. Analysis of the distribution of this block of genes showed that most of the STs in the herds that were either thought to be free of SD or where the disease status was unclear did lack them, but this also commonly was the case for the isolates in STs in the herds with a history of SD (Table 4). Indeed, the relative distribution of isolates with plasmid types 1 and 7 was not significantly different between the herds from the three reported health statuses. If these genes were essential for virulence it would have been expected that they were more likely to be present in herds where disease has been reported, which they were not. One possible interpretation is that although they are not essential, their activity may tend to increase the likelihood of successful colonization by *B*. *hyodysenteriae* to levels where lesions and/or disease starts to occur [14]. Isolates belonging to ST150 were present in a breeding herd and in six production herds connected to it, only one of which had reported disease. These isolates had all six plasmid genes, but it cannot be excluded that they were defective in other genes or gene expression that reduced their ability to cause disease [14]. Herds 5 and 63, where disease was not observed, each had isolates of two different STs, and in both cases one had plasmid type 7 and the other had plasmid type 1.

Substantially more isolates from the 1980s and 1990s possessed all or most of the plasmid genes than did isolates from the early 2000s and 2014/16 (Table 5), and there were highly significant differences over this period between the number of isolates with plasmid types 1 and 7 (Table 6). In the case of the three STs that were present both in 2014/16 and in previous decades, two had plasmid type 7 in the earlier decades and plasmid type 1 in 2014/16, again supporting an evolutionary trend to lose these genes. If these genes are important in facilitating colonization this might explain the apparent tendency for the reported occurrence and severity of SD in Australia to have been reduced in recent years, despite the spirochete being widespread. This conclusion is tempered by the fact that relatively small numbers of isolates were examined, and that the isolates recovered from the 1980s and 1990s were from diagnostic samples recovered from herds with disease. Nevertheless, the isolates from the early 2000s and some of the isolates from 2014/16 also were from herds where SD had been reported. Ultimately it will be important to test both the current and historic Australian isolates in a standardized experimental infection model to evaluate their potential to colonize and cause disease.

From the trends observed it seems likely that the full plasmid was present in the strains originally introduced into Australia, and that genes gradually have been lost from the plasmid in different strains over the last 40 years. Indeed, three isolates from 2014/2016 did not have the plasmid at all. Loss of the plasmid genes by the spirochete suggests that they are not essential for viability, and indeed it may be that a loss of these genes improves and selects for survival by making the spirochete less likely to proliferate to levels that provoke inflammation and disease: in turn this would result in less antimicrobial usage to eliminate the infection. It was interesting that two of the isolates lacking the whole plasmid also were weakly hemolytic. Where collections of suitable isolates are available in other countries, studies are required to determine whether similar temporal changes in plasmid gene content and phenotype of *B*. *hyodysenteriae* strains have occurred.

The methodology used for detection of susceptibility to the three key antimicrobials used to control SD in Australia was selected to mirror that used in earlier studies, and so allow temporal comparisons of trends. It was recognized that more robust definition could have been achieved had additional antimicrobials and antimicrobial dilutions been used, but this was not the main objective of the study. The antimicrobial susceptibility patterns observed were variable, even amongst isolates with the same ST, and even where these were isolated from the same herd. For example, ST150 contained multi-resistant isolates as well as others that were susceptible to one or more of the antimicrobials tested. In this case, assuming that an otherwise identical strain was present in all the herds, it could have come under different selection pressures depending on antimicrobial exposure.

Resistance to lincomycin was substantially more common than amongst the Australian isolates collected 10–15 years earlier. An increasing trend for resistance to lincomycin also has been recorded in Australian isolates collected before 2002 [54]. As is found worldwide, resistance of *B*. *hyodysenteriae* isolates to tylosin was almost universal. The point mutation that causes tylosin resistance at position 2058 in the 23S rRNA gene is known also to increase the MICs for the lincosamide antibiotics [55], and if this occurred it also might help to explain the trend for the increased MICs to lincomycin that were found.

Resistance of *B*. *hyodysenteriae* isolates to tiamulin is an increasing problem in a number of European countries, and where it occurs it severely reduces options for control of SD [56]. In an analysis of Australian isolates collected before 2002 none were resistant to tiamulin [54], but in this study isolates belonging to four of the 30 STs (13.3%) from 2014/16 were resistant to tiamulin, with 59% showing intermediate susceptibility. This was substantially more common than amongst Australian isolates from 2006–2007, but only slightly more common than amongst isolates from 2002–2006 (Table 7). This difference may reflect the origins of the isolates: ten of the 11 resistant isolates from 2002–2006 originated from herds in Queensland, but isolates from only two Queensland herds were obtained in the 2014/16 study. The single resistant isolate from the 2006–2007 set came from Victoria, and all four of the resistant isolates from 2014/16 also came from Victorian herds (the same State where most of the sampled herds were located). These isolates also were resistant to lincomycin and tylosin (ie they were multi-drug resistant), and three of them were recovered from herds that reported having SD. Multi-resistant isolates of this type represent a significant threat to the pig industry. Given the increasing trend in resistance to these drugs, it is important that detailed information is obtained about the susceptibilities of isolates present in individual herds before control measures are implemented. Equally where susceptible isolates are present consideration should be made to eradicate the infection using the drugs that are still effective.

## Conclusions

This study has demonstrated that *B*. *hampsonii* and *B*. *suanatina*, the newly described agents of SD in North America and Europe, are unlikely to be present in Australia. On the other hand the classical agent *B*. *hyodysenteriae* is relatively common and widespread. The strains that are currently circulating generally are different from those found in the past, are less likely to carry the block of six virulence-associated plasmid genes, and are different from strains from other countries. Evidence was found for the likely transmission of strains between piggeries that were epidemiologically linked. More of the recently isolated strains showed antimicrobial resistance than in the past, and the identification of four different multi-drug resistant strains was concerning. An important finding was the identification of *B*. *hyodysenteriae* in a large number of herds that had no disease, or only mild disease of previously unknown etiology.
